# Fabrication of novel nitrogen-doped δ-Al_2_O_3_ NPs: characterization and performance evaluation for electrocatalytic degradation of organic dyes

**DOI:** 10.1039/d6na00277c

**Published:** 2026-06-08

**Authors:** Elbadawy A. Kamoun, Ahmed T. Mosleh, Habiba A. Hossni, Tarek A. Yousef, Nourhan A. M. Ragab, Heba Y. Zahran, V. Ganesh, Mohamed Hafez, Ibrahim S. Yahia, Shereef A. Fareed

**Affiliations:** a Department of Chemistry, College of Science, King Faisal University Al-Ahsa 31982 Saudi Arabia ekamoun@kfu.edu.sa badawykamoun@yahoo.com +966-201283320302; b Nanotechnology Section, Egyptian Company for Carbon Materials El-Sheraton/El-Nozha Cairo Egypt; c Chemistry Department, College of Science, Imam Mohammad Ibn Saud Islamic University (IMSIU) Riyadh 11623 Saudi Arabia; d Laboratory of Nano-Smart Materials for Science and Technology (LNSMST), Department of Physics, Faculty of Science, King Khalid University P. O. Box 9004 Abha Saudi Arabia dr_isyahia@yahoo.com; e Faculty of Engineering and Quantity Surveying, INTI-IU Nilai Malaysia; f Faculty of Management, Shinawatra University Pathum Thani Thailand; g National Research Institute of Astronomy and Geophysics (NRIAG) Helwan 11421 Cairo Egypt

## Abstract

Herein, we report the construction of a novel N-doped δ-Al_2_O_3_ nanoparticle (NAO)-based gelatin support using a sol–gel/auto-combustion method. XRD analysis proved the NAO NPs' tetragonal structure, which confirmed the delta-phase (*δ*) formation of Al_2_O_3_ NPs. SEM micrographs of NAO NPs showed irregular shapes with rocky surfaces and grain sizes ranging from 34.9 to 66.6 nm. The direct band gap energy of the fabricated NAO-3 NPs was approximately 5.305 eV, indicating a promising response to electrocatalytic activity. NAO-3 NPs demonstrated effective electrocatalytic degradation of both carmine and eosin yellow dyes. The fabricated NAO-3 NPs catalyst achieved degradation efficiencies of ∼96.3% (0.28112 min^−1^) and 97.5% (0.19828 min^−1^) within 10 min and 12 min, respectively, for carmine and eosin yellow dyes, while mixed dyes were degraded by ∼98.60%. Trapping analysis revealed that the primary species that initiated the electrocatalytic degradation of carmine dye was the O_2_˙^–^ radical. After five cycles of electrocatalytic degradation, the electrocatalytic performance of NAO-3 NPs decreased slightly to 90.5%, demonstrating that NAO NPs possess excellent stability and recyclability throughout the electrocatalytic reaction process for the degradation of organic pollutants.

## Abstract 2

Schematic showing the electrocatalytic degradation of organic dyes.

## Graphical abstract

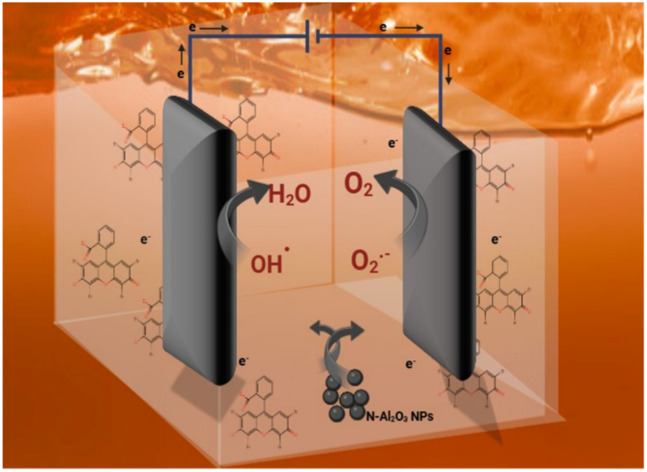

## 1. Introduction

Human activity rapidly depletes freshwater due to chemical pollution, leading to socioeconomic issues, environmental degradation, and food insecurity.^1^ To eradicate this pollution, scientists are searching for new approaches to address environmental contamination.^2^ In particular, synthetic dyes pose a significant risk to our water supplies.^3^ However, wastewater treatment involves physical, biological, and chemical processes supported by analytical methods for the dynamic control and process management of hazardous pollutants, which are essential for safeguarding the environment against pollution.^4,5^ On the other hand, toxic pollutants in wastewater from textiles,^6^ printing,^7^ and food processing pose environmental hazards.^8^ Dyes are hazardous to the environment because of their toxicity, stability, and vibrant colors.^9^

Carmine dye is a naturally occurring anionic organic dye derived from the dried corpses of female *Coccus cacti* L. insects; it is soluble in alkaline solutions and stable at pH values above 6.^10^ It is commonly utilized in cosmetics, as a natural colorant, and in the plastics and pharmaceutical fields, and it has a special role in the textile industry.^11^ Eosin yellow (EY) is a water-soluble fluorescein, an anionic dye created from xanthene, and is used in industrial aquatic environments and applications such as painting, printing, and leather.^12,13^ However, it could lead to human genotoxicity, irritate the skin, and block protein interactions; thus, purification is an essential issue.^14,15^

Numerous techniques for treating wastewater have been investigated, including oxidation, adsorption, biodegradation, coagulation, flocculation, photodegradation, and electrocatalytic degradation, leading to either partial or complete mineralization of organic components.^16,17^ Among other methods, electrocatalysis is the basis of the advanced oxidation process (AOP). Electrochemical advanced oxidation processes (EAOPs) have received outstanding consideration for their competent degradation of organic pollutants to reduced or inorganic substrates *via* generating highly active hydroxyl radicals (˙OH).^18,19^ Over the past decade, electrochemical (EC) treatment methods, such as electrodeposition, electro/flotation, electrocoagulation, and electro-oxidation, have generated considerable interest because of their low cost and excellent flexibility, making them strong candidates for treating industrial wastewater.^20,21^

EC oxidation has numerous advantages, including mild operation, environmental friendliness, selective degradation, stable long-term results, and a wide range of applications associated with electrocatalytic degradation for lowering water pollution and generating green energy.^22,23^ Various scientific organizations have widely employed EC oxidation methods, including direct and indirect oxidation, for removing wastewater-containing dyes by enhancing the catalytic activity of catalyst materials while controlling operating conditions.^24–26^ EC treatment requires active groups with sufficient oxidative qualities or electrolyte solutions supplied *via* various pathways, including hydroxyl radicals, sulfate radicals, and chlorine radicals, either by applying energy or using a catalyst.^27,28^ Thus, redox processes, which are facilitated by reactive oxygen species (ROS), can degrade a range of organic compounds, including dyes, into environmentally beneficial chemicals.^16,18,29^ Various semiconductor metal oxides with different morphologies have been explored as a sustainable solution for converting contaminants into simpler substances due to their high surface charge and chemical inertness, such as Al_2_O_3_, CuO, TiO_2_, and Fe_2_O_3_.^30^ This method is inexpensive and crucial for light absorption, charge transfer, and hydrogen production.^31,32^ Alumina (Al_2_O_3_) is an extremely important ceramic material for theoretical research and practical applications.^33^ It has ideal electrical insulation, toughness, erosion and corrosion resistance, and durability.^34^ Because of its accessibility and reconfigurable architecture, Al_2_O_3_ is being investigated as a possible metal oxide with high durability and dye removal rates.^30,35^ Al_2_O_3_ NPs play a significant role in the formation of energy band structures and generate oxidative species, thus functioning as an electrocatalytic degradation entity during application studies.^36^ Using the sol–gel method, low-temperature, energy-efficient, and readily scaled-up production approaches have been reported elsewhere.^37^ The sol–gel auto-combustion method was chosen because it is economical, straightforward, and useful, resulting in a low calcination temperature and good product homogeneity.^38^ Dopant elements are added to bare semiconductors to create energy between the valence and conduction bands, which appropriately alters the semiconductors' light-absorbing characteristics. N-doped semiconductors appear to be the most favorable dopant elements, and they are active when exposed to electrocatalytic activity.^39^

Numerous studies have used Al_2_O_3_ NPs as a catalyst for the adsorption of cationic dyes, underscoring their potential to alleviate water pollution, as previously studied by Manikandan *et al.*,^40^ where γ-alumina (Al_2_O_3_) nanoparticles were synthesized using Tween-80 and formamide. Methylene blue dye was successfully adsorbed from water by Al_2_O_3_ NPs, exhibiting a capacity increase ranging from 490 to 2210 mg g^−1^ as the starting concentration increased from 50–400 mg L^−1^ under conditions of pH 9 and 10 min reaction time at 60 °C.^40^ For instance, Pang *et al.* reported a 97.96% degradation efficiency for RGO/ZnO compared to Congo red dye in 60 min.^41^ Ghozza *et al.* used Bi_0.9_Ba_0.1_FeO_3_ for degrading 96.5% of Congo red dye during 4 min.^42^ Xu *et al.* prepared g-C_3_N_4_/Fe_3_O_4_/AlON *via* a hydrothermal method. Under visible light, g-C_3_N_4_/Fe_3_O_4_/AlON exhibited a significant enhancement of the degradation efficiency for methyl orange (MO), achieving 95.9% degradation within 240 min.^43^

In this study, we have fabricated N-doped δ-Al_2_O_3_ NPs using a sol–gel/auto-combustion method. The as-fabricated materials were characterized by XRD, FTIR, TGA, SEM, energy-dispersive X-ray analysis (EDX), and diffuse reflectance spectroscopy (DRS) to determine the crystalline nanostructures, chemical structure, surface morphologies, and compositions. Optical properties were evaluated by various analyses. Nitrogen doping was introduced as an innovative approach to modify the electronic structure of Al_2_O_3_ NPs, effectively reducing their band gap, enhancing charge carrier mobility, and promoting electron transfer efficiency during electrocatalytic activity and stability, and the electrocatalytic degradation of organic chemicals, *e.g.*, carmine and eosin yellow dyes, was investigated using novel N-doped δ-Al_2_O_3_ NPs.

## 2. Materials and methods

### 2.1. Materials

Aluminum acetate (C_2_H_5_O_4_Al, 99%), gelatin (purity: 98%), carmine dye (C_22_H_20_O_13_), eosin yellow (EY) (C_20_H_6_Br_4_Na_2_O_5_), sodium chloride (NaCl, purity 98), sodium nitrate (NaNO_3_, purity 98%), ethylene diamine tetraacetic acid (EDTA) (C_10_H_16_N_2_O_8_, purity 98%), ascorbic acid (C_6_H_8_O_6_, purity 99%), and isopropyl alcohol (C_3_H_8_O, purity 99%) were obtained from Sigma-Aldrich, Germany. Distilled water (DW) was used for further experiments.

### 2.2. Fabrication of N-Al_2_O_3_ NPs

**Fig. 1:**
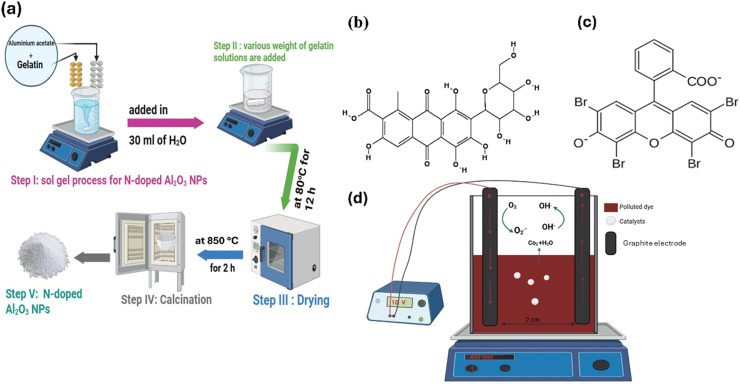
(a) Schematic of fabrication of N-doped δ-Al_2_O_3_. (b) Structure of carmine dye and (c) eosin yellow dye. (d) Schematic of used electrocatalytic cell.

N-doped δ-Al_2_O_3_ NPs were synthesized by a sol–gel/auto-combustion method.^44^ To 5 g of aluminum acetate, gelatin with different ratios (0.001, 0.01, 0.1, 0.25, 0.5, 1, 5, and 10 g) was added as a nitrogen source to create N-doped δ-Al_2_O_3_, with sample codes NAO-1, NAO-2, NAO-3, NAO-4, NAO-5, NAO-6, NAO-7, and NAO-8, respectively, based on the incorporated gelatin content. Then, 30 mL of distilled water was added and mixed well at 25 °C with continuous stirring to obtain a homogeneous solution that formed a gel. The gel was dried at 80 °C in an oven for 12 h, and calcined for 2 h at 850 °C to produce N-doped δ-Al_2_O_3_ NPs, as shown in Fig. 1(a).

The reaction mechanism for the synthesis of N-doped δ-Al_2_O_3_ NPs is as follows:1
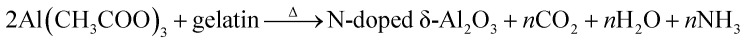


### 2.3. Study of electrocatalytic degradation

Electrocatalytic degradation was employed in a single electrochemical cell at ambient temperature. Two parallel graphite electrodes, with an area of 1.40 mm^2^ and a distance of 2 cm between the cathode and anode, were fixed in the applied cell, and the solution conductivity was 0.063 S m^−1^. Next, 200 mL of carmine (100 ppm) and eosin yellow (50 ppm) dyes were merged with 0.05 g L^−1^ of the NAO NPs catalyst at pH = 7, and 10 mL of 1 M NaCl was then added to improve the conductivity. The carmine and EY dyes were degraded using a regulated DC power supply. The applied potential (DC) remained at 10 V during the electrochemical process, as shown in Fig. 1(d). The voltage operates the electrochemical process and particle repolarization at the electrode. The particle at the electrode becomes more repolarized as the voltage rises; the electrochemical reactions of the carmine and EY dyes change, thus the degradation effect improves, and the reaction speed increases.^45^ The samples were taken at specific times using similar absorption wavelengths for UV-Vis spectrophotometry analysis. The percentage of dye degradation was determined using the following equation:^46^2
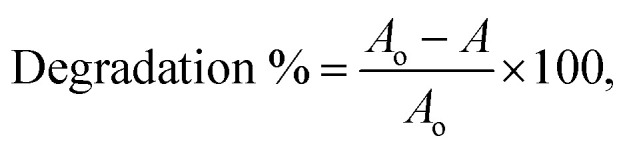
where *A*_o_ is the initial absorbance of the dye, and *A* is the absorbance at different times.

### 2.4. Instrumental devices

XRD patterns were recorded using a diffractometer (Shimadzu LabX-XRD-6000, Japan) operating in the reflection mode with CuK (radiation) (*λ* = 1.5406 Å). Data were collected at a scanning rate of 0.02° s^−1^ over a range of 10–80° at 30 kV and 30 mA. The crystalline structures of the prepared materials were determined from the diffraction patterns, and the XRD peaks were indexed using X'pert High Score software.

FT-IR spectroscopy (Nicolet IS50, Thermo Electron Corporation, Waltham, MA) was used to determine the chemical composition and functional groups of the prepared samples at 4000–400 cm^−1^.

The microstructure of the composites was examined at 30 kV by SEM (JSM-6360, JOEL, Japan), with energy-dispersive X-ray spectroscopy (EDX); the elemental mapping technique was used for elemental composition analysis, and the Image J program was utilized for determining grain size.

HR-TEM (Jeol, JEM-2100 plus, Japan) was utilized to examine the nanocomposites and photocatalysts at an accelerating voltage of 200 kV.

A Shimadzu UV-3600 UV-Vis DRS instrument (Japan) was used for optical scattered reflectance measurements in *λ* (200–800 nm). KBr served as the reference material, combined with the integrating sphere attachment.

Surface area analysis was performed *via* N_2_ adsorption–desorption measurements for sample NAO-3 NPs at 77 K using a BET surface area and pore size distribution analyzer (BELSORP-miniX 10115, Microtrac, Inc., USA).

A UV-Vis single-beam spectrophotometer (LISCO-GmbH, Germany) was employed to follow the degradation process under ambient conditions.

## 3. Results and discussion

### 3.1. XRD analysis

**Fig. 2:**
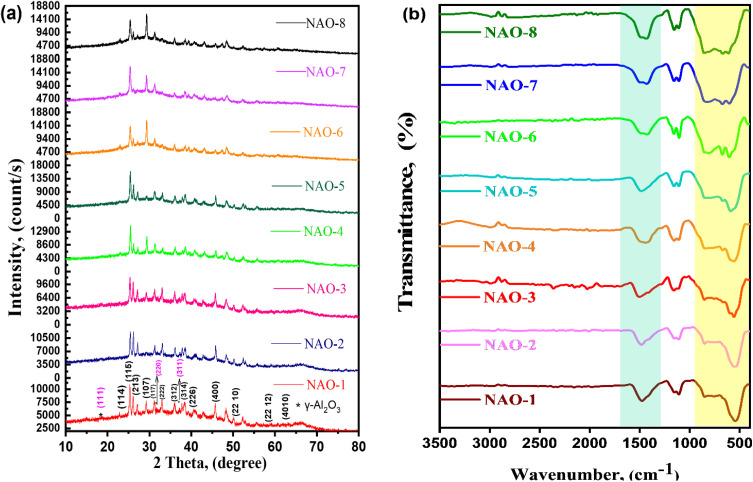
(a) XRD patterns for N-doped δ-Al_2_O_3_ NPs. (b) FTIR spectra for N-doped δ-Al_2_O_3_ NPs.

Fig. 2(a) presents diffraction patterns obtained by XRD analysis of the N-doped δ-Al_2_O_3_ NPs crystalline structure, which proves the delta-phase (*δ*) of δ-Al_2_O_3_, matching the standard card no. (ICDD 00-016-0394).^47^ XRD patterns show peaks at 2θ angles of ∼22.8°, 25.17°, 26.11°, 29.04°, 31.20°, 33.19°, 35.86°, 38.42°, 40.22°, 45.70°, 49.99°, 57.19°, and 62.30°, conforming to crystal planes (1 1 4), (1 1 5), (2 1 3), (1 0 7), (1 1 7), (2 2 2), (3 2 1), (3 1 4), (2 2 6), (4 0 0), (2 2 10), (2 2 12), and (4 0 10), respectively. N-doped δ-Al_2_O_3_ NPs demonstrate a tetragonal structure with dimensions *a* = *b* = 7.944 Å, *c* = 23.120 Å, *α* = *β* = *γ* = 90°. The Al_2_O_3_ NPs displayed another phase, gamma (*γ*) γ-Al_2_O_3_, which matches the standard card no. (ICDD 10-0425) with a clear cubic structure.^48^ The absence of additional peaks indicates the high purity of the N-doped δ-Al_2_O_3_ NPs sample. The peaks further indicate that both have the same crystal structure and that the precursor-to-fuel ratio is irrelevant to the crystal structure. Despite not affecting the structure, the fuel quantity influences the unit cell volume.^41^Equation (3) shows the Debye–Scherrer formula, which was used to verify the average crystallite size:3
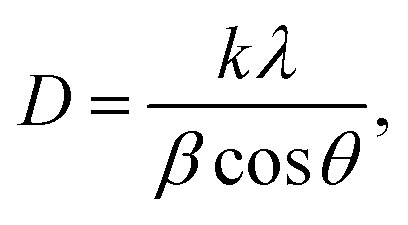
where *λ* is wavelength in nanometers, *β* is the full width at half maximum of the peak, *θ* is Bragg's angle (degrees), *D* is the crystallite size, and *k* is Scherrer's constant (*k* = 0.9).

4 *δ* = 1/*D*^2^

5
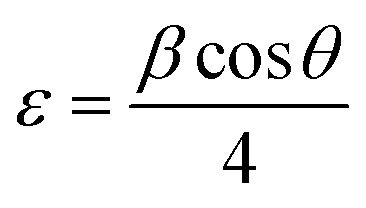


Eqn (4) and (5) are used to estimate the micro-strain (*ε*) and average dislocation density (*δ*) for N-doped δ-Al_2_O_3_ NPs.^49^

From Fig. 2(a), the peak is narrow, indicating the remarkable crystallinity of NAO NPs. The lattice structure of the tetragonal structure can be evaluated using eqn (6), as shown in Table 1.6
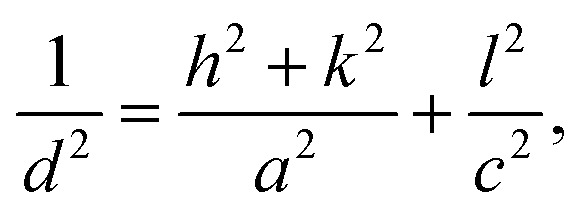
where (*D*) is the size of crystallite NAO NPs using X-ray diffraction, (*θ*) is Bragg's angle, (*λ*) is the wavelength of X-ray radiation (1.5406 Å), and (*β*) is the full width at half maximum (FWHM) of the diffraction peak. Incorporating gelatin in the preparation of NAO nanoparticles notably influences their structural characteristics. As a polymerization agent and growth terminator, gelatin is crucial in reducing the particle size to around 20 nm. XRD analysis revealed that the presence of gelatin affects the crystallite size and significantly enhances the crystallinity of NAO NPs, resulting in more uniformly distributed and well-defined structures.

**Table 1:** Dislocation density and average crystal size, micro-strain, and lattice structure values were calculated using XRD spectra for NAO NPs

Samples	Average *D* (nm)	(*δ*) (nm)^−2^	Lattice strain	*d*-spacing (Å)	*a* = *b* (Å)	*c* (Å)	*V* (Å^3^)
NAO-1	21.38	2.829 × 10^−3^	1.744 × 10^−3^	1.9843	7.937	23.179	1460.28
NAO-2	22.69	2.783 × 10^−3^	1.768 × 10^−3^	1.9803	7.921	23.155	1452.88
NAO-3	21.25	2.910 × 10^−3^	1.770 × 10^−3^	1.9830	7.932	23.159	1456.72
NAO-4	22.05	2.556 × 10^−3^	1.669 × 10^−3^	1.9796	7.798	23.119	1405.84
NAO-5	19.76	3.366 × 10^−3^	1.917 × 10^−3^	1.9800	7.920	23.123	1450.42
NAO-6	21.30	2.843 × 10^−3^	1.750 × 10^−3^	1.9898	7.959	23.115	1464.24
NAO-7	20.311	2.858 × 10^−3^	1.798 × 10^−3^	1.9799	7.919	23.119	1449.81
NAO-8	21.01	3.184 × 10^−3^	1.817 × 10^−3^	1.9810	7.924	23.102	1450.57

### 3.2. FTIR analysis

FT-IR spectroscopy was used to evaluate the absorption of IR radiation by the sample. The obtained infrared spectrum reflects the relationship between the absorption bands (vibrational bands) and the chemical components of the sample.^50^ The functional groups in N-doped δ-Al_2_O_3_ containing different ratios of gelatin were determined from the FTIR spectra, as shown in Fig. 2(b). N–H stretching is detected at *ν* 3347 cm^−1^.^51^ The gelatin absorption bands are found in the infrared spectra of the amide band region.^52^ The bands in the *ν* 1687 cm^−1^ area indicate the presence of the C=O bond.^53^ The C=O stretching mode corresponds to the bands at *ν* 1153 and 1106 cm^−1^.^54^ The peaks at *ν* 545 cm^−1^ and 753 cm^−1^ are attributed to aluminum oxide stretching.^35^ The peaks at *ν* 545 cm^−1^ shifted to a higher *ν* of 611 cm^−1^, and the intensity of these peaks reduced as the amount of gelatin increased.

### 3.3. TGA analysis

**Fig. 3:**
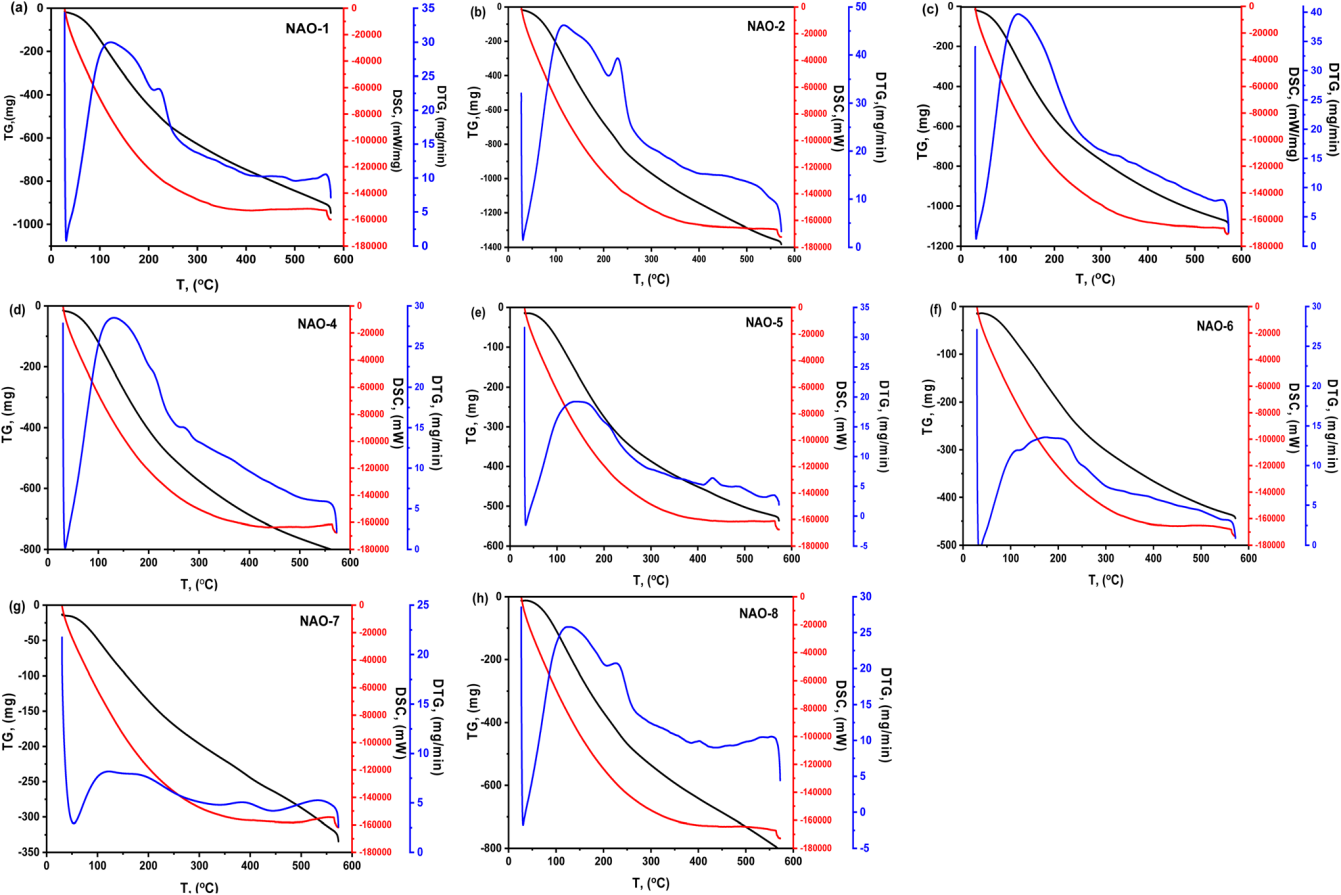
(a–h) TGA, DSC, and DTG thermographs of N-doped δ-Al_2_O_3_ NPs coded (NAO-1 to NAO-8), respectively.

TGA is frequently used to investigate the residual mass at higher temperatures and the thermal stability of nanocomposites. Fig. 3(a–h) illustrate the TG, DSC, and DTG curves of the NAO NPs sample. The sample's mass decreased as the temperature rose from RT to 550 °C, according to TG and DSC data. The sample loses moisture, humidity, or adsorbed water between 25–100 °C, and the weight of NAO NPs gradually decreases between 12–18% at 550 °C.^55^ The weight loss changes become more obvious as the amount of gelatin increases during the synthesis step.^56^ The DTG curve shows little change in the exothermic degradation between room temperature and 350 °C. Water evaporation caused a tiny endothermic thermometric peak at 200 °C. The breakdown reaction's primary temperature range is 400–500 °C, where there is a noticeable endothermic peak.^57^ The most obvious mass change occurred at 404 °C. Thereafter, the rate of mass change gradually decreased with temperature.

### 3.4. FE-SEM and EDX investigation

**Fig. 4:**
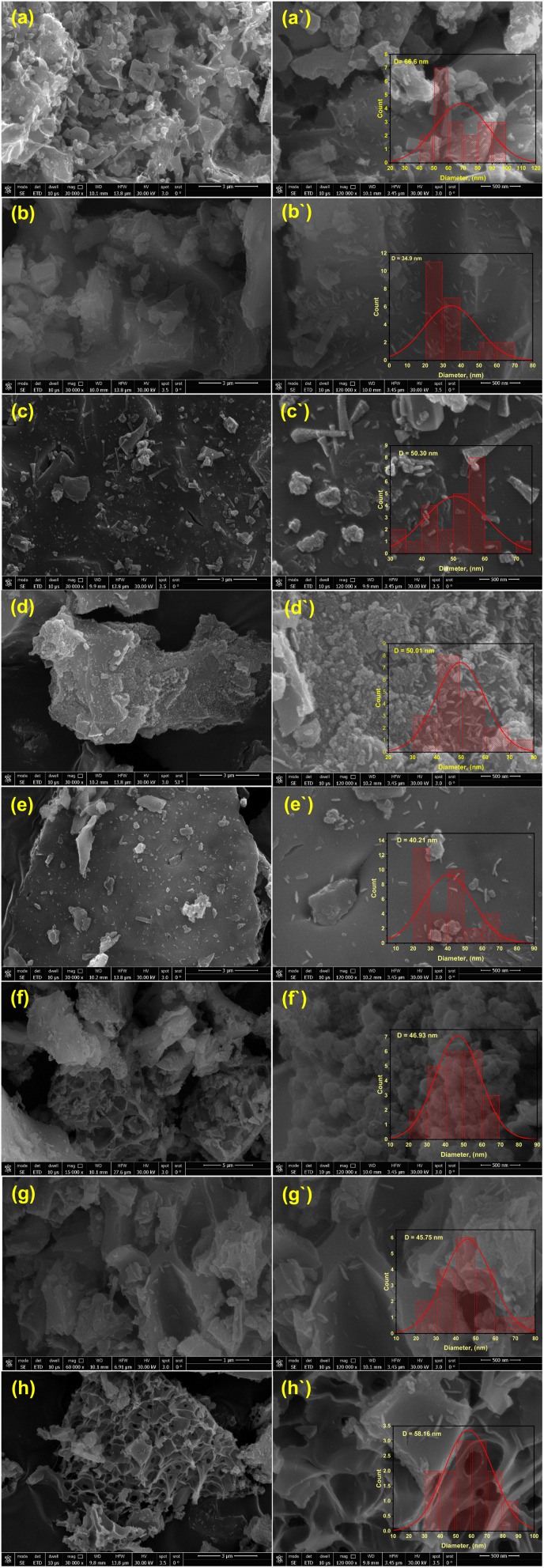
(a–h) SEM micrographs of N-doped δ-Al_2_O_3_ NPs; all samples were imaged at (3 µm, 30k× and applied voltage 30 kV). (a′–h′): samples were imaged at (500 nm, 120k× and applied voltage 30 kV) and their histogram of particle size .

**Fig. 5:**
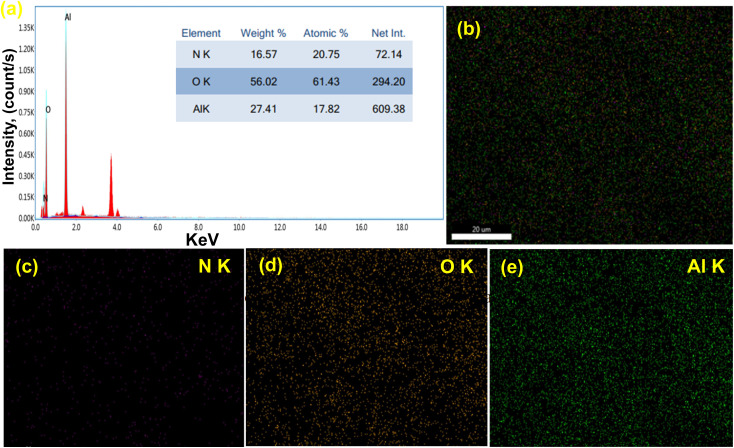
(a) EDX of NAO-3 NPs and (b–e) elemental mappings of N-doped δ-Al_2_O_3_ NPs.

FE-SEM was used to determine the surface morphological alterations and microstructure of the NAO NP samples. Fig. 4(a–h) show crystalline structures that are semi-spherical and irregular with varied sizes and shapes; clusters of many NPs are also visible in the images.^58^ Tadic *et al.* investigated the causes of the variations in particle shape and cluster formation in relation to the magnetic characteristics of NPs, which influence the particle size and shape.^59^Fig. 4(a′–h′) show that the size distribution of NAO NPs is in the range of 34.9–66.6 nm. Fig. 5(a) shows energy-dispersive X-ray spectroscopy (EDX) results for the prepared NAO NPs. The visible peaks are attributed to Al, O, and N without any impurities. A trace amount of element Au was also found because the sample was coated with gold during sample preparation before the FE-SEM investigation to make it conductive.^60^ Based on the EDX spectra, the weight percentages of Al, O, and N were 27.41, 56.02, and 16.57%, respectively. Fig. 5(b–e) display the elemental mapping of NAONPs, confirming homogeneous element distribution.

### 3.5. HR-TEM analysis

**Fig. 6:**
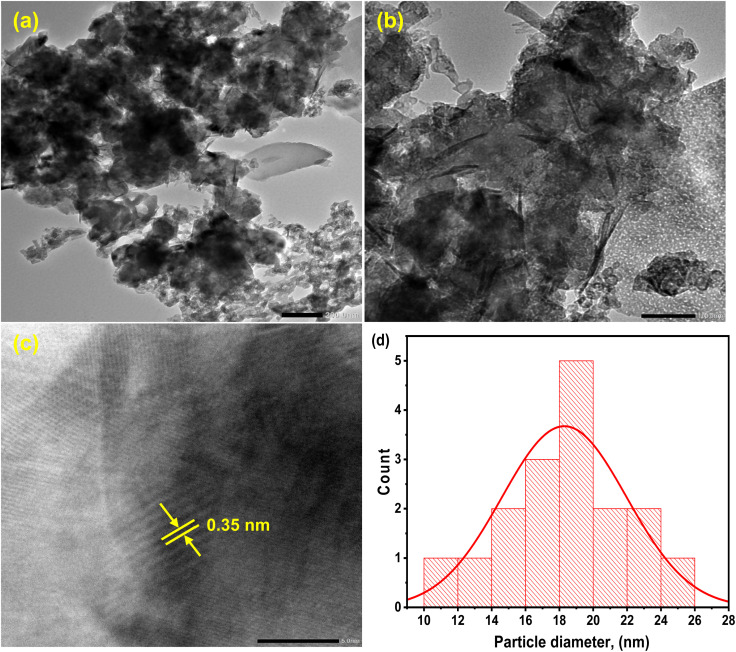
(a and b) TEM images of N-doped δ-Al_2_O_3_ NPs, (c) HR-TEM image, and (d) particle size distribution histogram.

Fig. 6(a and b) show the morphology of NAO-8 NPs, as determined by TEM investigation. The images show that NAO-8 NPs have an irregular layered structure with mesoporous properties, and are randomly dispersed. This observation is similar to results reported by Manikandan *et al.*^40^ The images also show that the prepared NAO-8 NPs have uniformly sized particles and are less agglomerated. Furthermore, the crystalline interplanar lattice spacing of 0.35 nm precisely matches the (115) crystal plane of Al_2_O_3_ NPs, as shown in the HR-TEM image in Fig. 6(c). Fig. 6(d) shows that the average particle size of NAO-8 NPs is 18 nm.

### 3.6. UV-Vis DRS analysis

**Fig. 7:**
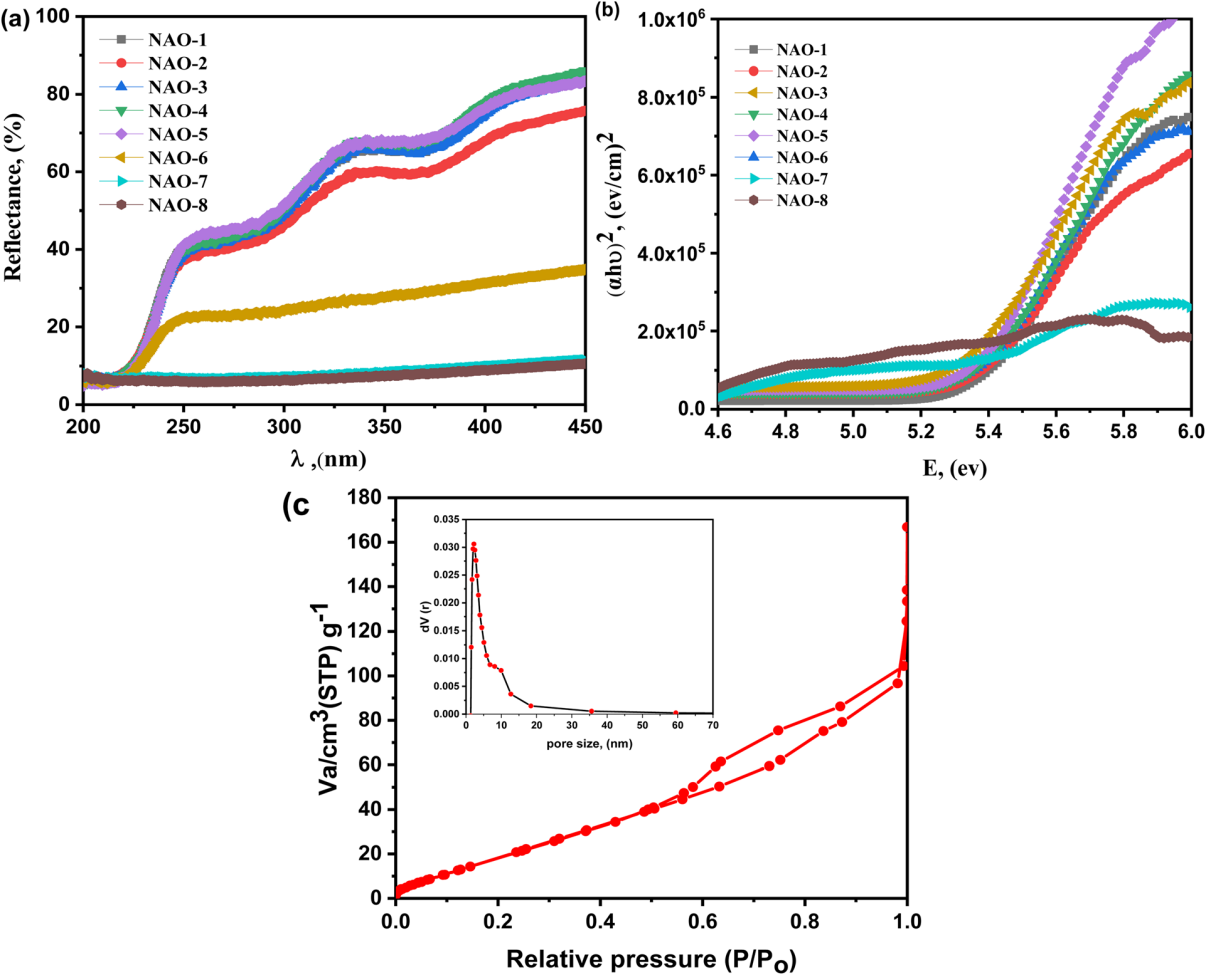
(a) Reflectance spectra, (b) direct energy band gap of N-doped δ-Al_2_O_3_ NPs, and (c) BET analysis of NAO-3 NPs, involving surface area analysis, total pore volume, and average pore diameter.

**Fig. 8:**
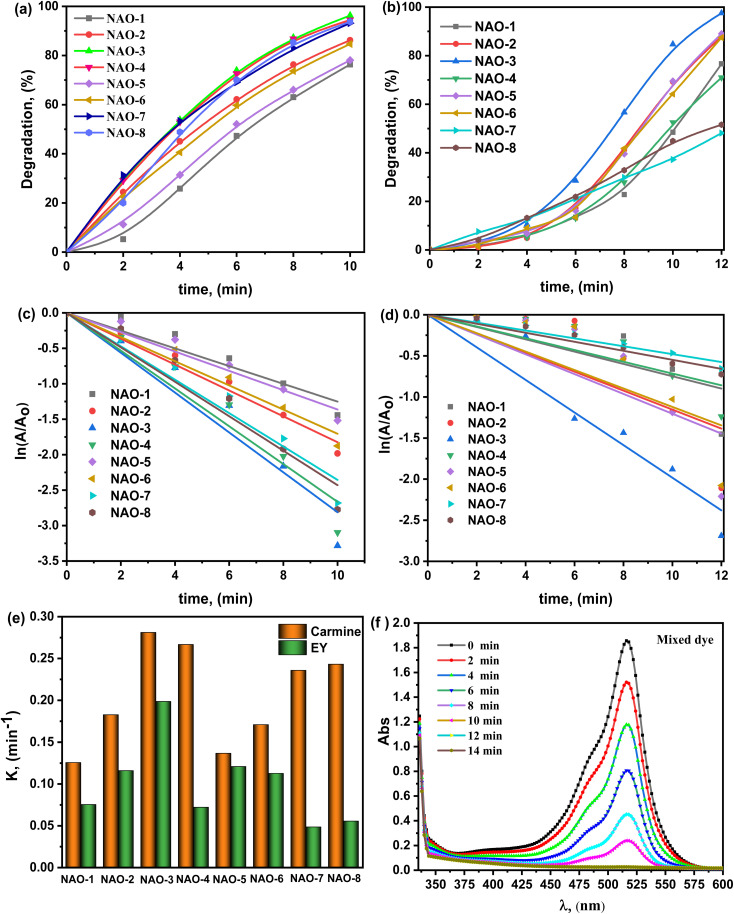
(a and b) Degradation efficiencies of carmine and eosin yellow dyes, (c and d) degradation kinetics, (e) reaction rate constants for the degradation of carmine and eosin yellow dyes with different NAO NPs, and (f) UV-Vis spectra of the mixed dye with NAO-3 NPs.

DRS was conducted to obtain information on the optical properties of N-doped δ-Al_2_O_3_ NPs. Fig. 7(a) presents the spectra obtained from the UV-Vis DRS analysis of N-doped δ-Al_2_O_3_ NPs samples; energy band gaps and certain *λ* edge absorption wavelengths of the samples were determined. The Kubelka–Munk function F(*R*) is relative to the absorption coefficient of NAO NPs and was calculated from eqn (7):^61^7
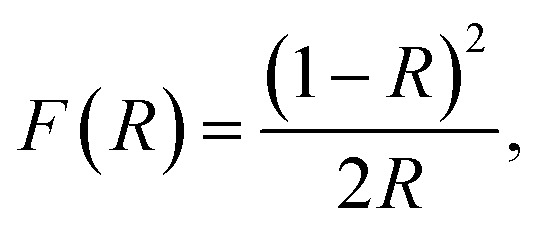
where *R* is the reflectance of NAO NPs. The results show that the absorption of the fabricated NAO NPs is in the 450–200 nm range. As shown in Fig. 8(a), a noticeable weak and broadened absorption band was observed at 221 nm in all the prepared N-doped δ-Al_2_O_3_ NPs samples because of the charge transfer inter-band transition from the O^2−^ valence band to the Al^3+^ conduction band^62^ of the four-fold coordinated surface. The absorption peak experienced a red shift as the amount of gelatin increased. In the UV spectrum, NPs only exhibited a weak capacity to absorb light.^63^ Due to charge transfer transitions from O_2_^−^ to Al^3+^ centers, oxygen-induced surface defect variations, along with crystal phase transformations, lead to crystalline δ-Al_2_O_3_ with varied morphologies. The absorption peak at 279 nm in Fig. 7(a) is due to threefold-coordinated O^2−^ anions on the surface of the as-fabricated N-doped δ-Al_2_O_3_ NPs.^64^ To estimate the optical energy gap of N-doped δ-Al_2_O_3_ NPs, Tauc's equation was used as follows:^65^

8 (*αhν*)^*n*^ = *A*(*hν* − *E*_g_),

where *hν*, *α*, *A*, and *E*_g_ are incident light frequency, absorption coefficient, an energy-independent constant, and band gap energy, respectively. Furthermore, the value of (*n*) is determined by the kind of optical transitions in semiconductors. It was set to 1/2 for a direct bandgap semiconductor and 2 for an indirect bandgap semiconductor. Fig. 7(b) shows a direct bandgap. The computed *E*_g_ values of N-doped δ-Al_2_O_3_ NPs with varying gelatin concentrations are presented in Table 2. The direct band gap energy value of the fabricated NAO-3 was approximately 5.305 eV. This observation is similar to results reported by Akouibaa *et al.*,^66^ where the material exhibits semiconductor characteristics despite its insulating behavior because of surface defects that add energy levels to the band gap, making it easier to generate charge carriers and providing them with possible catalytic applications.^67,68^

**Table 2:** Direct bandgaps corresponding to N-doped δ-Al_2_O_3_ NPs

Samples	*E*_g_ direct (eV)	Electrocatalytic degradation of carmine dye		Electrocatalytic degradation of eosin yellow dye	
		Degradation (%)	*K* (min^−1^)	Degradation (%)	*K* (min^−1^)
NAO-1	5.382	76.35	0.12515	76.61	0.07484
NAO-2	5.348	86.22	0.18242	87.85	0.11542
NAO-3	5.305	96.25	0.28112	97.50	0.19828
NAO-4	5.340	94.48	0.26653	71	0.07156
NAO-5	5.361	78.08	0.13628	89.02	0.12043
NAO-6	5.332	84.69	0.17051	87.47	0.11215
NAO-7	5.328	93.15	0.23562	48.09	0.04796
NAO-8	5.355	93.75	0.24303	51.58	0.05486

### 3.7. BET (Brunauer, Emmett and Teller) analysis

N_2_ adsorption–desorption isotherms were used to clarify the specific surface texture and pore size of the NAO-3 NPs sample. The results demonstrate type IV behaviour with a type H3 hysteresis loop, according to IUPAC classification, across the relative pressure range *P*/*P*_0_ ≈ 0–1, as shown in Fig. 7(c). Low adsorption in the low-pressure area and enhanced adsorption with expanding pressure are characteristics of type IV isotherms.^69^ From BET surface area analysis, total pore volume, and average pore diameter were found to be 85.078 m^2^ g^−1^, 0.1704 cm^3^ g^−1^, and 8.0138 nm, respectively.

### 3.8. Electrocatalytic activity study

The electrocatalytic degradation of carmine and EY dyes over the N-doped δ-Al_2_O_3_ NPs catalyst was assessed under ambient conditions and atmospheric pressure at an applied potential (DC) of 10 V. To realize an adsorption/desorption equilibrium, the catalyst-loaded solution of dye was maintained in a dark environment for half an hour. For 12 minutes, the solution was exposed to a continuously applied DC voltage to drive the electrocatalytic process over the NAO NPs catalyst. Every two minutes, a sample was taken. The highest absorption of carmine dye was observed at *λ*_max_ 518 nm, and at *λ*_max_ 515 nm for EY dye. The electrocatalytic degradation of these dyes in the absence and presence of NAO NPs was completed in 10 min for carmine dye and 12 min for EY dye, as shown in Fig. S1 and S2 (SI). There was a decrease in the intensity of the absorption band with irradiation time for carmine and EY dyes in the presence of NAO NPs, which revealed that nanocomposite NAO NPs effectively degraded the dyes. The electrolysis of carmine and EY dyes was 71.7% and 59.1%, while the maximum electrocatalytic degradation of carmine and EY dyes with NAO-3 NPs was 96.25% and 97.50%, respectively, as shown in Fig. 8(a and b).

#### 3.8.1. Kinetics study

A kinetics study was conducted to achieve a more quantitative understanding of the electrocatalytic activity of NAO NPs. The pseudo-first-order kinetics study yielded the rate constant of the electrocatalytic reaction based on eqn (9):9
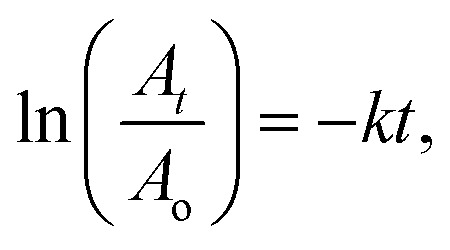
where *A*_o_, *A*_*t*_, and *K* represents the initial absorbance, the absorbance at time *t*, and the kinetic constant at any point during the degradation process (min^−1^), respectively.^70^ The rate constant was determined from the plot of 
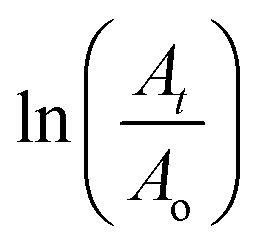
 as a function of time, which showed a linear correlation, as presented in Fig. 8(c and d). The electrocatalytic behavior of the fabricated NAO NPs catalyst was investigated by varying the incorporated gelatin content while maintaining a constant dye solution concentration. The rate constant *k* for carmine and EY dyes is shown in Table 2. Rate constants were calculated as 0.28112 and 0.19828 min^−1^ with NAO-3 NPs for the electrocatalytic degradation of carmine and EY dyes, as shown in Fig. 8(e). NAO-3 NPs have high electrocatalytic activity because introducing gelatin to δ-Al_2_O_3_ significantly reduces energy band gaps, and a high surface area, which readily absorbs photons to increase the final efficiency and improve electrocatalytic activity, compared to other reported findings in Table 3. N-doped metal oxides might also lower the energy band gap of aluminum oxide NPs. With abundant N atoms, gelatin is an ideal nitrogen precursor that improves the characteristics of δ-Al_2_O_3_ NPs by raising the conductivity of aluminum oxide particles or lowering their contact resistance.^71,72^Table 3 presents a comparison of many electrocatalysts used for the degradation efficiency of eosin yellow and carmine dyes.

**Table 3:** Comparison of different electrocatalysts for the degradation efficiency of eosin yellow and carmine dyes

Type of catalyst	Method of preparation	Reaction condition							Ref.
		Dye	Dye conc. (ppm)	Catalyst conc. (g L^−1^)	Electrolyte	Current voltage (V)	Time (min)	Degradation (%)	
N-Fe_2_O_3_/G	Auto-combustion method	IC	100	0.05	1 M NaCl	5	45	95.18	73
50% BiOCl/TiO_2_	Microemulsion process	EY	50	1	0.10 M Na_2_SO_4_	—	120	93.6	74
Graphite-ZnO NCs	Sol–gel method	EY	70	—	0.1 M Na_2_SO_4_	1.5	120	76	75
BaO/G	Auto-combustion method	MB	10	0.05	1 M NaCl	10	25	97.1	76
NAO-3 NPs	Auto-combustion method	CarmineEY	100	0.05	1 M NaCl	10	10	96.25	Present study
			50				12	97.50	

#### 3.8.2. Electrocatalytic degradation of mixed dyes

Fig. 8(f) displays the range of two-dye combinations (carmine and EY) and shows degradation over time. The wavelength overlap for EY and carmine at 516 nm was found to be the maximum absorbance value for each dye. Because of their similar π-conjugated electronic structures and anionic chromophores, carmine and EY dyes absorb light in almost the same visible *λ* range, which causes spectral interference or a mixed color appearance.^77,78^ In the dye-degradation study, the NAO-3 NPs electrocatalyst was chosen and applied. After 14 minutes, there was a decrease in each dye's absorption intensity under electrocatalytic activity without complete degradation. The degradation of the mixed dye was 98.60%. Compared to anionic dye pollutants, NAO-3 NPs are more efficient in eliminating dyes because the surface of NAO-3 NPs contains Lewis acid sites (Al^3+^ ions) that degrade these dyes. The results show that NAO-3 NPs electrocatalysts can be used efficiently in wastewater treatment.

#### 3.8.3. Influence of radical scavenger species

**Fig. 9:**
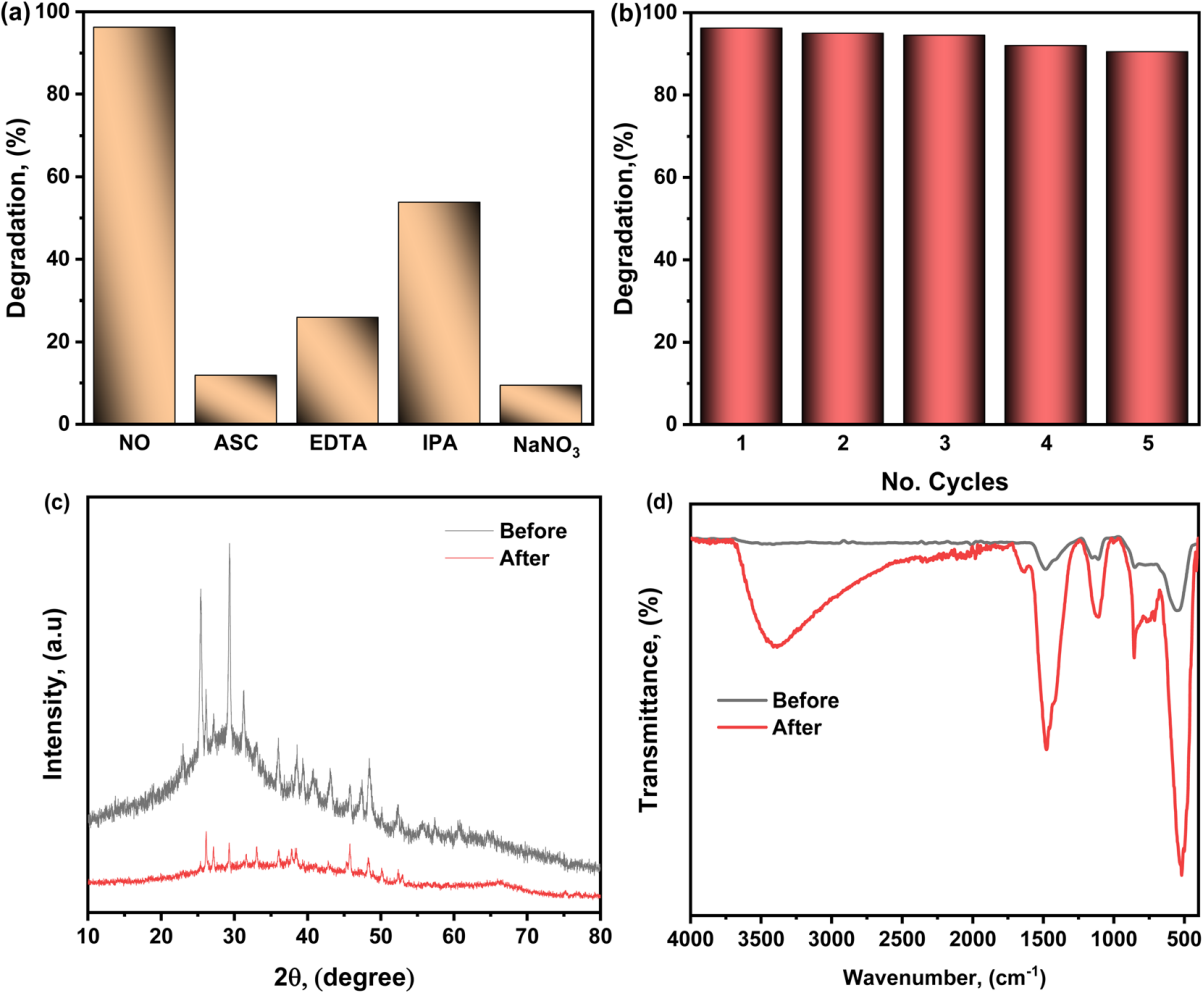
(a) Electrocatalytic degradation of carmine dye in the presence of NaCl and different scavengers. (b) EC degradation of carmine dye for five runs, (c) XRD patterns, and (d) FTIR spectra after reusability.

To investigate the role of ROS in the NAO-3 NPs electrocatalyst, scavengers were introduced to quench the active species responsible for photodegradation, as shown in Fig. 9(a). Ascorbic acid, isopropyl alcohol, ethylenediaminetetraacetic acid (EDTA), and sodium nitrate (NaNO_3_) were previously used as scavengers of O_2_˙^–^, OH˙, h^+^ and e^−^, respectively, for EC reactions.^79^ The degradation rate of carmine dye was reduced upon the addition of exact trapping-scavengers from 96.3% with NAO-3 NPs. This decrease in carmine dye degradation is ascribed to the specific trapping scavengers, which removed approximately 11.6%, 53.7%, 25.7% and 9.2% of carmine dye, respectively. In Fig. 9(a), the degradation efficiency of carmine dye doesn't significantly decrease with the addition of isopropyl alcohol and EDTA. Additionally, the introduction of ascorbic acid and NaNO_3_ resulted in a substantial reduction in the degradation efficacy of carmine dye. These phenomena suggest the crucial roles of O_2_˙^–^ and e^−^ in the degradation of carmine dye.

#### 3.8.4. Mechanism of the electrocatalytic oxidation of carmine dye degradation

The removal of pollutants through electrochemical oxidation can be achieved through direct anodic oxidation, which results in poor decontamination, or indirect oxidation, due to molecules forming on the anode surface. These two processes can occur simultaneously or independently in a reactor.^80^ In electrochemical oxidation, electrolytes, such as sulfates, nitrates, perchlorates, and chlorides, greatly improve electrical conductivity and lower energy consumption, impacting the removal of contaminants and the generation of active species. In contaminated wastewater, chloride is a common electrolyte that produces active species such as active chlorine compounds to form chlorine gas (Cl_2_), which then reacts with water to make HOCl; HOCl then breaks down into hypochlorite ions (ClO^−^), a powerful oxidant that is responsible for dye degradation *via* the following reactions:^81–83^

10 2Cl^−^ → Cl_2_ + 2e^−^ (anode)

The hypochlorous acid releases chlorine and then dissociates to produce the hypochlorite ion.

11 Cl_2_ + H_2_O → H^+^ + Cl^−^ + HOCl

12 HOCl ↔ H^+^ + ClO^−^

13 Dye + ClO^−^ → Cl^−^ + CO_2_ + H_2_O (cathode)

Electrons in NAO-3 NPs are driven from the valence band to the conduction band by an applied potential, producing free electrons (e^−^) and holes (h^+^). These electrons cause dissolved oxygen to transform into superoxide (O_2_˙^–^) radicals. First, electrons go through an external circuit during degradation processes, producing very reactive superoxide radicals. Our research has demonstrated that carmine dye degradation on NAO-3 NPs is primarily caused by O_2_˙^–^ and e^−^.^84^ Interestingly, O_2_˙^–^ is mainly liable for degrading carmine dye on NAO-3 NPs, as shown in Fig. 9(a). The equations below describe the formation of active species on the catalyst surface:^85^

14 O_2_ + e^−^ → O_2_˙^–^

15 O_2_˙^–^ + H_2_O → OH^−^ + HO_2_˙

16 HO_2_˙ + H^+^ → OH^−^ + OH˙

17 OH^−^ + h^+^ → OH˙

18 OH˙ + carmine dye → CO_2_ + H_2_O + other product

#### 3.8.5. Reusability and stability

For real-world uses, the electrocatalyst's stability is crucial; five cycling tests were conducted using the same experimental setup, with ten minutes of exposure to the applied voltage, to examine the stability of NAO-3 NPs. Centrifugation was used to collect the catalyst after each cycling experiment, and it was then cleaned with distilled water for use in the subsequent run. For real-world uses, the electrocatalyst's stability and reusability are crucial. Fig. 9(b) illustrates how NAO-3 NPs degrade carmine dye; after five cycles of electrocatalytic degradation, the NAO-3 NPs' electrocatalytic performance decreased slightly from 96.25% to 90.5%, demonstrating the NAO-3 NPs' excellent stability and recyclability throughout the electrocatalytic reaction process. Additionally, the unavoidable loss of electrocatalyst weight during washing and centrifugation may be responsible for the small decline in electrocatalytic performance. The structural stability of NAO-3 NPs after five cycles was investigated using XRD and FTIR analysis. Fig. 9(c) shows no obvious shift in the characteristic diffraction peaks compared to the XRD patterns of fresh NAO-3 NPs, indicating that the crystalline structure remains after repeated cycles. A slight decrease in the intensity of XRD peaks is due to a reduction in crystallinity or exposure of active planes after recycling.^86^Fig. 9(d) shows FTIR spectra, where the absorption of water is responsible for the broad band absorption at *ν* 3422 cm^−1^.^87^

## 4. Conclusions

A novel N-doped δ-Al_2_O_3_ NPs was fabricated by a sol–gel/auto-combustion method, where gelatin was incorporated into δ-Al_2_O_3_ NPs as a source for N-doping. The phase structure, chemical structure, morphology, and elemental composition were verified using different instrumental characterization methods. DRS analysis showed that the incorporation of gelatin into δ-Al_2_O_3_ reduced the optical bandgap energy. Thus, N-doped δ-Al_2_O_3_ NPs were used to study the electrocatalytic activity for the degradation of carmine, eosin yellow, and mixed dyes. The chosen NAO-3 NPs catalyst notably achieved degradation efficiencies of 96.25%, 97.5%, and 98.60% in 10, 12, and 14 min for carmine, eosin yellow, and mixed dyes, respectively. The reactive species responsible for carmine dye degradation showed that O_2_˙^–^ and HOCl, with H_2_O and Cl^−^ oxidation to Cl_2_ as a primary oxidizing agent, were mainly responsible for degrading carmine dye on NAO-3 NPs. After assessing the results, the chosen and preferred NAO-3 NPs catalyst showed good efficiency and recyclability following rescue cycles.

## Ethical approval

This article does not contain any animal studies by the authors.

## Author contributions

A. T. M., H. Y. Z. and T. A. Y.: methodology, formal analysis, and data curation. H. A. H. and N. A. M R.: resources, data optimization, software, data analysis. M. H. and S. A. F.: resources and project management. E. A. K. and I. S. Y.: project management, supervision, wrote the original draft and reviewed the final draft. All authors approved the current and final version of the manuscript for submission.

## Conflicts of interest

The authors declare that they have no known competing financial interests or personal relationships that could have appeared to influence the work reported in this paper.

## Supplementary Material

NA-008-D6NA00277C-s001

## Data Availability

The data sets used and/or analyzed during the current study are available from the corresponding authors upon reasonable request. Supplementary information (SI) is available. See DOI: https://doi.org/10.1039/d6na00277c.
