# Supplementary material for: Fabrication of novel nitrogen-doped δ-Al2O3 NPs: characterization and performance evaluation for electrocatalytic degradation of organic dyes
Source: Nanoscale Adv. 2026 Jun 8;8(14):4033–48. doi: 10.1039/d6na00277c (PMC13267191; doi:10.1039/d6na00277c)
Supplement: NA-008-D6NA00277C-s001 [file NA-008-D6NA00277C-s001.pdf]

Supplementary Information (SI) for Nanoscale Advances.
This journal is © The Royal Society of Chemistry 2026

Supplementary materials

Fabrication of novel nitrogen-doped δ-Al_2O_3 NPs: Characterization and performance evaluation for electrocatalytic degradation of organic dyes

Elbadawy A. Kamoun[1*], Ahmed T. Mosleh[2], Habiba A. Hossni[2], Tarek A. Yousef[3], Nourhan A.M Ragab[2], Heba Y. Zahran[4], V. Ganesh[4], Mohamed Hafez[5,6], Ibrahim S. Yahia[4*], Shereef A. Fareed[7]

[1]*Department of Chemistry, College of Science, King Faisal University, Al-Ahsa 31982, Saudi Arabia.*
[2]*Nanotechnology Section., Egyptian Company for Carbon Materials, El-Sheraton/El-Nozha, Cairo, Egypt.*
[3]*Chemistry Department, College of Science, Imam Mohammad Ibn Saud Islamic University (IMSIU), Riyadh 11623, Saudi Arabia.*
[4]*Laboratory of Nano-Smart Materials for Science and Technology (LNSMST), Department of Physics, Faculty of Science, King Khalid University, P.O. Box 9004, Abha, Saudi Arabia.*
[5]*Faculty of Engineering and Quantity Surveying INTI, IU, Universi, Nilai, Malaysia.*
[6]*Faculty of Management, Shinawatra University, Pathum Thani Thailand.*
[7]*National Research Institute of Astronomy and Geophysics (NRIAG), Helwan 11421, Cairo, Egypt.*

**Corresponding authors: E.A. Kamoun, E-mail: ekamoun@kfu.edu.sa, Tel: 00201283320302, and I.S. Yahia. E-mail: dr_isyahia@yahoo.com.*

Supplementary materials

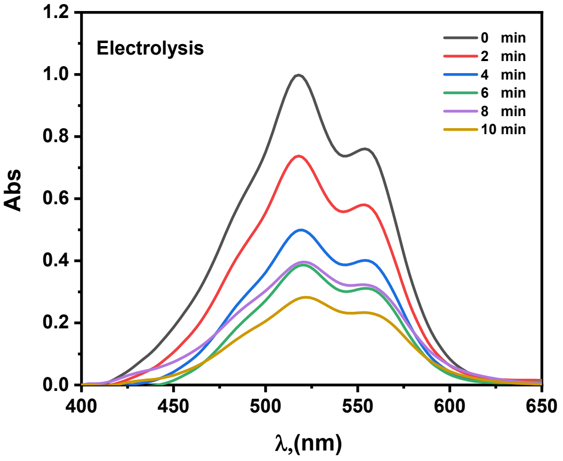

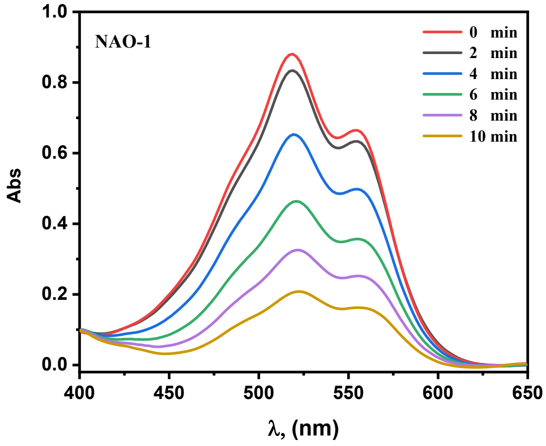

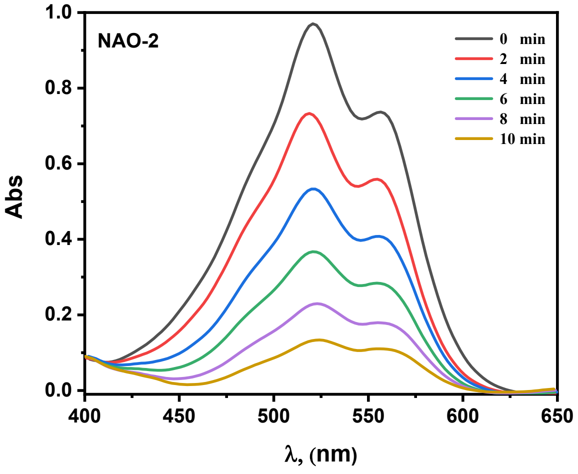

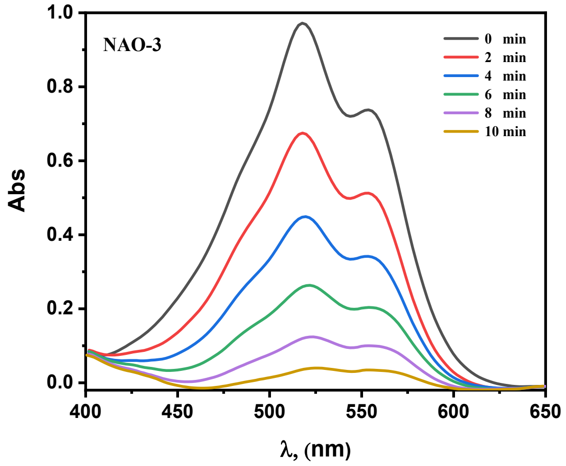

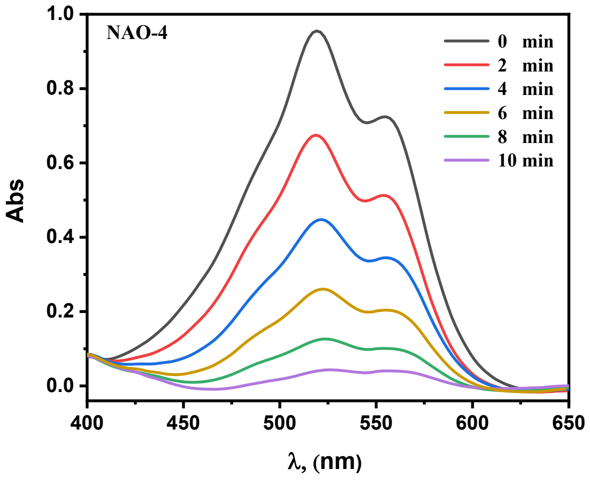

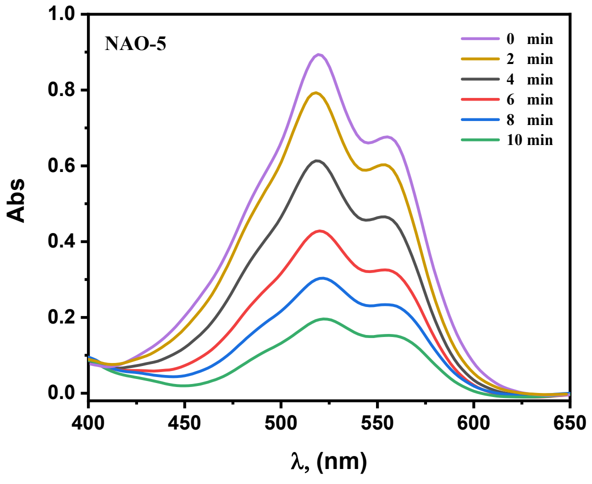

Supplementary materials

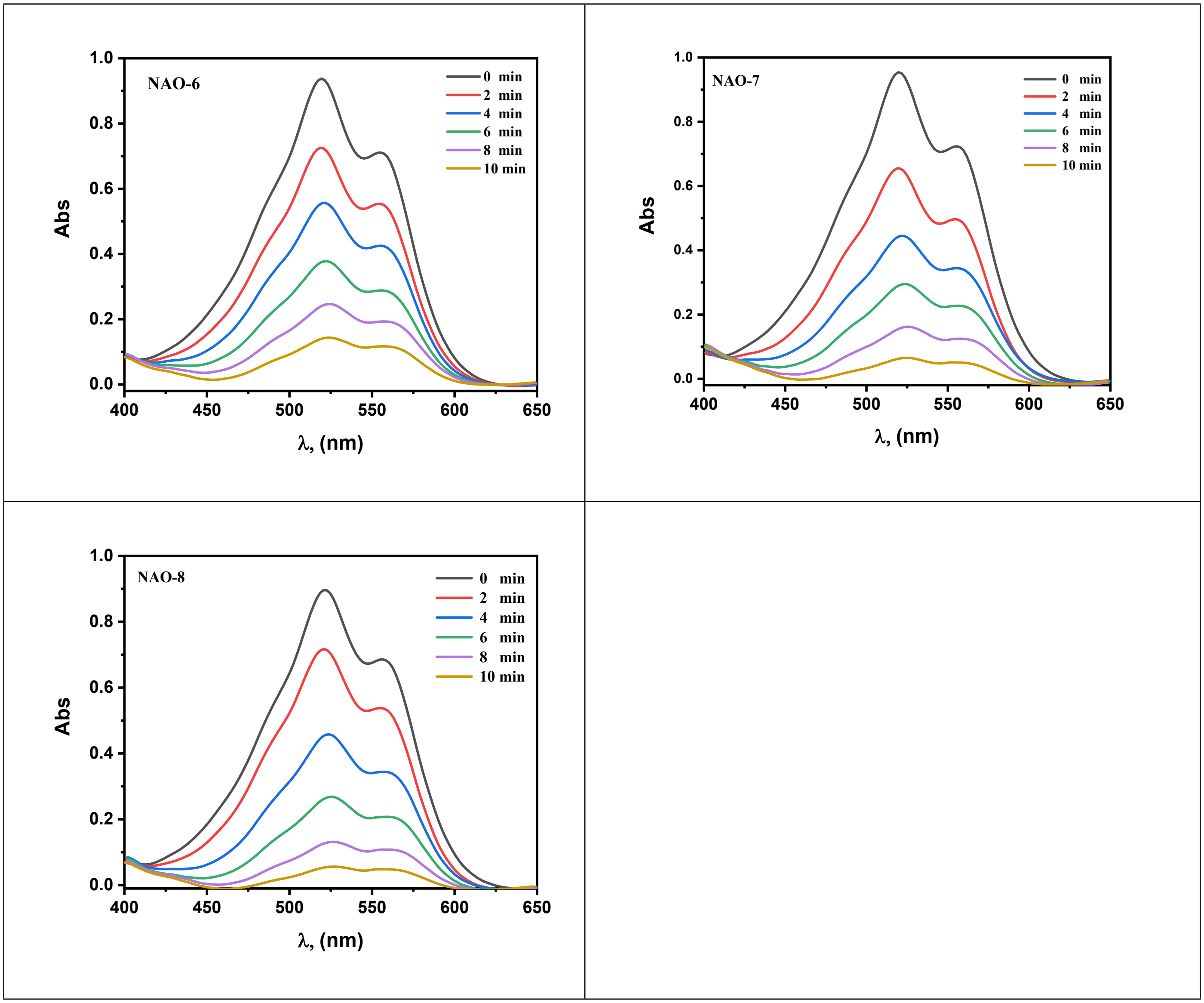

Fig. S1 UV–Vis spectra of *Carmine* dye with different NAO NPs.

Supplementary materials

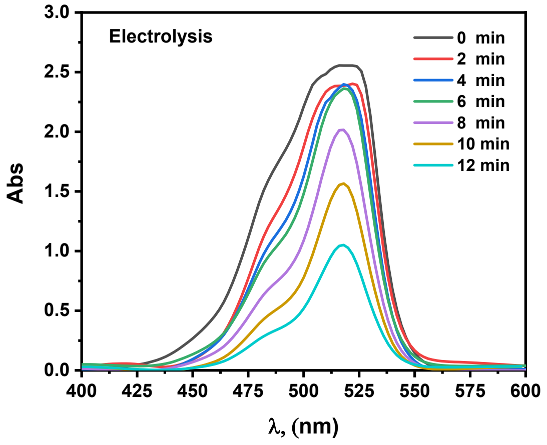

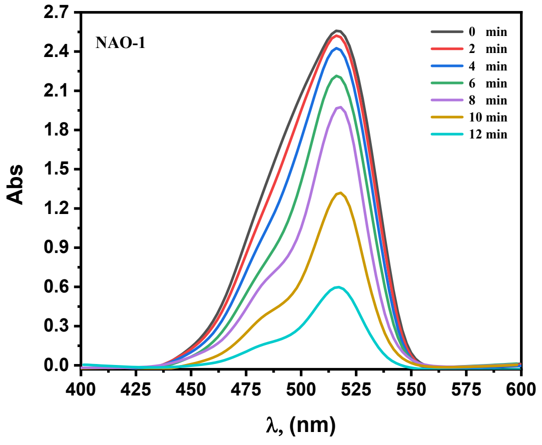

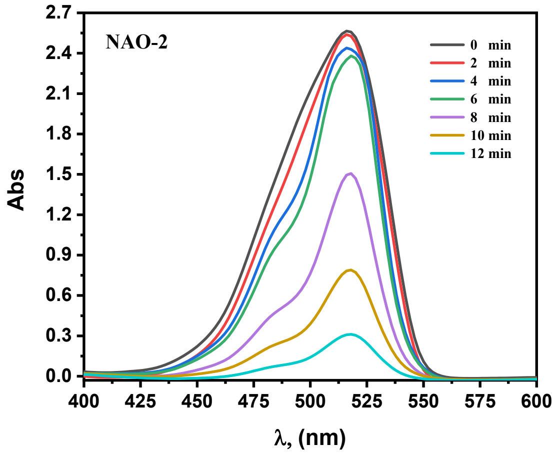

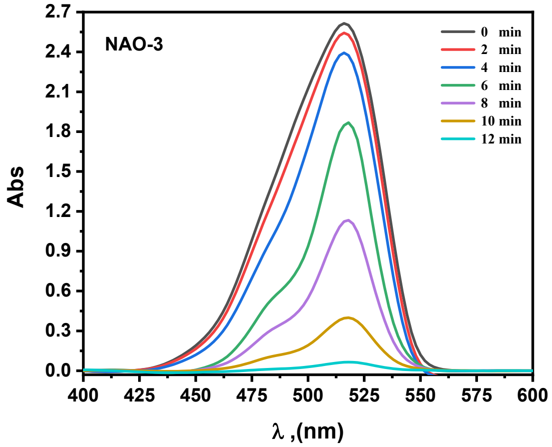

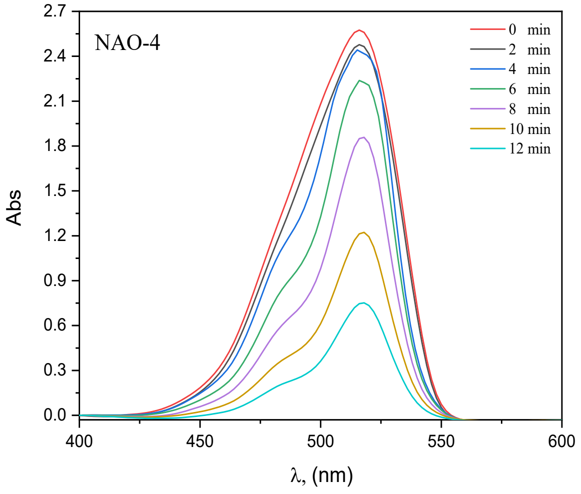

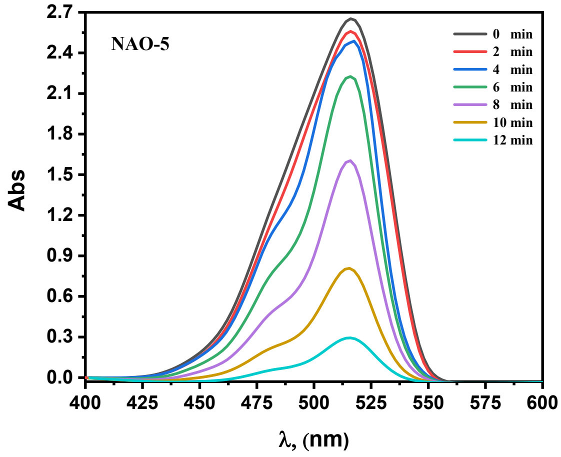

Supplementary materials

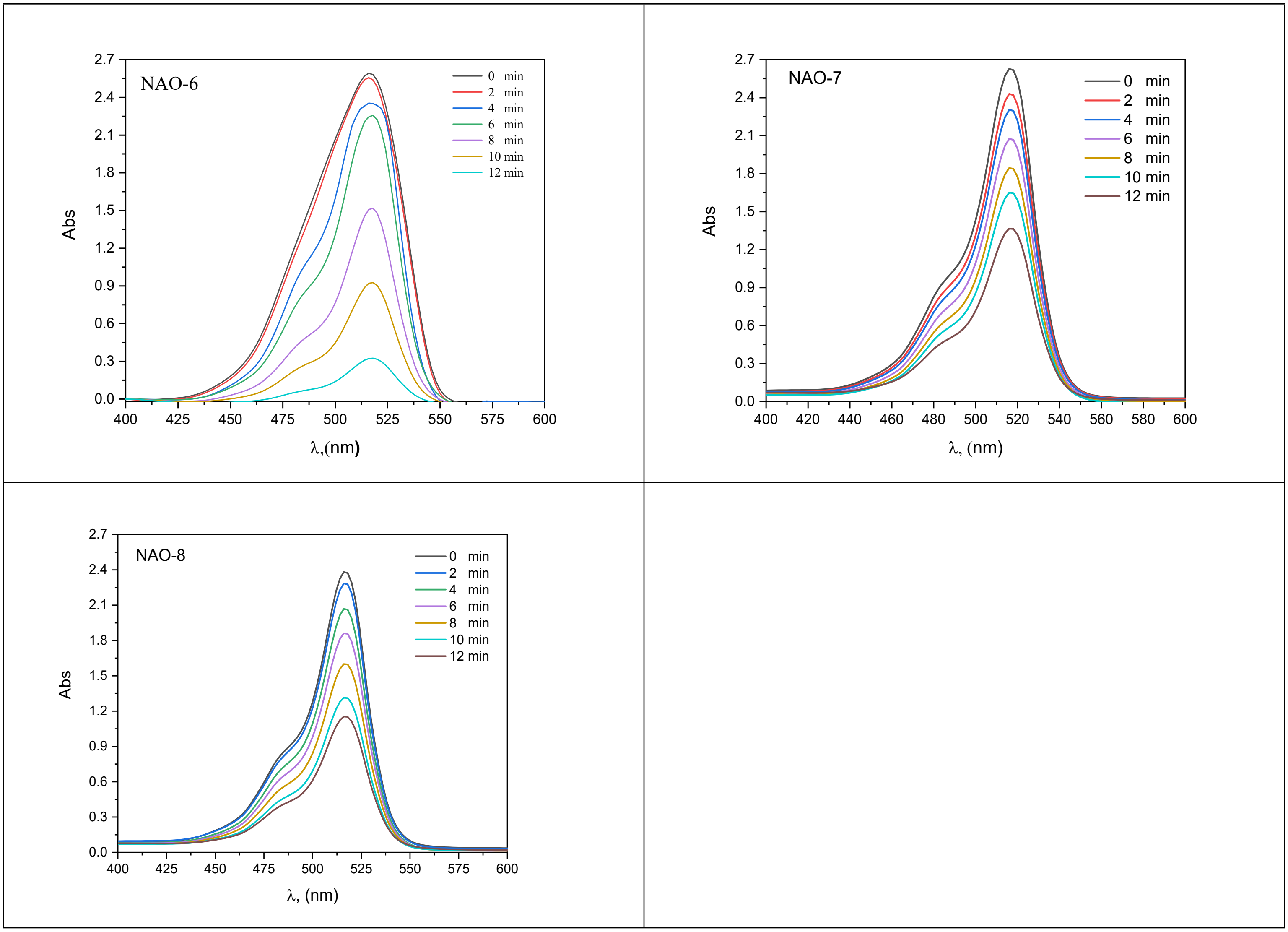

Fig. S2 UV–Vis spectra of *Eosin yellow* dye with different NAO NPs.
